# Modeling Pubertal Growth Variability in Schoolchildren From Florianópolis, Santa Catarina, Brazil: A Bayesian Analysis

**DOI:** 10.1002/ajhb.70129

**Published:** 2025-08-29

**Authors:** Luciano G. Galvão, Fábio C. Karasiak, Victor J. S. Conceição, Diego Augusto Santos Silva, Humberto M. Carvalho

**Affiliations:** ^1^ School of Sports Federal University of Santa Catarina Florianópolis Brazil; ^2^ Laboratory School, School of Education Federal University of Santa Catarina Florianópolis Brazil

**Keywords:** adolescence, longitudinal, maturation, SITAR

## Abstract

**Objective:**

This study aimed to describe inter‐individual variation in growth velocity during puberty among Brazilian children, considering maturity status to better understand differences in the timing and intensity of growth spurts.

**Methods:**

Longitudinal stature data from 398 Brazilian children (197 girls, 201 boys) aged 6–19 years, collected annually from 1997 to 2010, were analyzed. Growth and velocity curves were estimated using the SITAR model within a Bayesian framework. Maturity groups were classified based on the standard deviation of the estimated age at peak growth velocity, categorizing participants as early, average, or late maturers.

**Results:**

The mean age at peak growth velocity was 11.30 years for girls and 13.55 years for boys. Mean peak velocities were 8.38 and 9.52 cm/year, respectively. Pubertal takeoff occurred at 8.41 years for girls and 11.19 years for boys, with mean velocities of 5.85 and 5.43 cm/year. Early maturers exhibited earlier onset and higher growth velocities, while late maturers showed delayed growth for both sexes.

**Conclusions:**

Considerable variability in growth velocity patterns during puberty was observed. Compared to Northern Hemisphere populations, Brazilian children experienced earlier and more intense pubertal growth spurts. These findings highlight the importance of accounting for maturational timing in physical education planning, training load management, and pediatric growth assessment.

## 1 Introduction

Growth curves based on stature measurements are fundamental tools for interpreting patterns of physical growth during adolescence (Berkey et al. 1993). Longitudinal data are particularly valuable, as they allow for the estimation of key growth parameters such as peak height velocity (PHV) and the age at which it occurs (APHV) (Tanner et al. 1966). While general patterns of adolescent growth are well established, substantial inter‐individual and sex‐based variability persist (Cameron 2002). This variability is typically described in terms of timing—the age at which specific growth events like PHV occur—and tempo, which refers to the rate of progression through the growth spurt. A related dimension, intensity, captures the magnitude of growth velocity, particularly at its peak. Together, these parameters characterize individual growth trajectories and allow the description of between‐individual differences. This variation is of considerable interest but is seldom explored in detail in longitudinal studies, highlighting the need for analytical approaches capable of capturing and interpreting such differences (Iuliano‐Burns et al. 2001).

However, the majority of pubertal growth studies have been conducted in Northern Hemisphere populations (Kemper 2007; Malina et al. 1988), leaving a critical gap in data from other regions. In South America, longitudinal studies remain limited (Bogin et al. 1992; Johnston et al. 1976; Lopez‐Blanco et al. 1995; Mansukoski et al. 2020; Pereira et al. 2019), particularly in Brazil (Silva et al. 2019), despite the country's considerable genetic, cultural, and socioeconomic diversity (de Oliveira et al. 2022). This gap restricts our understanding of how regional, ancestral, and environmental factors influence growth patterns. The present study seeks to address this limitation by analyzing a unique longitudinal dataset from Florianópolis, Southern Brazil—a region with a predominantly European‐descendant population and relatively high socioeconomic indicators. While the dataset does not allow for formal testing of ancestry or socioeconomic effects, it provides a valuable reference for examining inter‐individual variation in pubertal growth within this specific regional and demographic context (Eveleth and Tanner 1991).

Accurate assessment of inter‐individual variation in the timing and tempo of adolescent growth spurts also presents methodological challenges. In many studies, lower age boundaries restrict the capacity to fully capture early maturers (Beunen et al. 1994; van Lenthe et al. 1996), while criteria for classifying maturity status often vary widely (Kozieł and Malina 2005), further complicating comparisons across studies.

To address these issues, this study applies the Super Imposition by Translation and Rotation (SITAR) model—a shape‐invariant, nonlinear multilevel approach that captures individual growth trajectories by modeling differences in size, timing, and velocity (Cole et al. 2010). Unlike simpler models, SITAR effectively describes the tempo and timing of growth, which are essential dimensions of pubertal development (Beath 2007; Lindstrom 1995).

Nevertheless, frequentist implementations of SITAR can encounter convergence problems, particularly with sparse or heterogeneous data (Simpkin et al. 2017). A Bayesian framework offers a flexible and robust alternative, accommodating the complexity, variability, and non‐linearity inherent in adolescent growth data (Gelman 2003; van de Schoot et al. 2014). This approach aligns with recent recommendations emphasizing the need for more nuanced and data‐adaptive models in this field (Boeyer et al. 2020).

Therefore, the aim of this study is to estimate the timing and intensity of pubertal growth in stature among Brazilian children using longitudinal data and a Bayesian SITAR model. Additionally, we describe inter‐individual differences in the timing and tempo of adolescent growth, contributing reference values for this regional population and offering insights that may support future research on the influence of ancestry and environmental factors on growth (Cameron 2002).

## 2 Methods

### 2.1 Study Design, Sample and Procedures

This study used retrospective data extracted from the database of the Colégio de Aplicação (CA), that is, the Laboratory School of the School of Education at the Federal University of Santa Catarina in Florianópolis, Brazil. CA is a public school serving students from elementary school (age 6) through high school (up to age 19). Student enrollment is determined by a public lottery, which promotes equal access and helps ensure that the student body reflects the ethnic and socioeconomic diversity of the broader Florianópolis population. Given its location in a heterogeneous urban center and its inclusive admission policy, CA provides a potentially representative sample of local adolescents.

Florianópolis, the capital of Santa Catarina in southern Brazil, had a population of 342,696 in 2000, with 96.2% residing in urban areas (Instituto Brasileiro de Geografia e Estatística—IBGE 2003). The city ranked sixth in Brazil on the Human Development Index (HDI) in the same year (Instituto Brasileiro de Geografia e Estatística—IBGE 2003). According to the 2000 census, 88% of residents aged 10–14 years identified as white (Instituto Brasileiro de Geografia e Estatística—IBGE 2003). The population of Santa Catarina is largely of European ancestry, particularly Portuguese, Italian, and German, which has shaped the region's demographics and culture (Instituto Brasileiro de Geografia e Estatística—IBGE 2011).

Annual longitudinal measurements of stature were collected between 1997 and 2010 as part of CA's physical education program. Trained physical education teachers followed standardized protocols (Nahas et al. 1992; Waltrick and de Fátima Duarte Silva 2000) and conducted assessments in the school's physical education laboratory. The dataset includes students aged 6 to 19 years.

In total, 3070 observations were included (girls: *n* = 1528; boys: *n* = 1542) from 398 students (girls: *n* = 197; boys: *n* = 201), based on annual repeated measurements. At study entry, participants were aged 6 to 9.9 years (girls) and 6 to 10.9 years (boys), with follow‐up extending to at least 14.0 years (girls) and 15.0 years (boys), and up to 19 years of age. To ensure adequate longitudinal coverage of the pubertal growth period, participants were included if they had a minimum of four repeated measurements within the age range of 9.9 to 14.0 years (girls) and 10.9 to 15.0 years (boys). The distribution of the number of measurements per participant is summarized in Table 1.

**TABLE 1:** Distribution of the number of measurements per participant.

	All sample	Female	Male
11 measurements	11	4	7
10 measurements	30	18	12
9 measurements	89	45	44
8 measurements	95	41	54
7 measurements	89	51	38
6 measurements	51	25	26
5 measurements	27	12	15
4 measurements	6	1	5

Chronological age and stature (cm) were retained, while maintaining student anonymity. Data access was granted following approval by the local ethics committee (80129124.0.0000.0121).

### 2.2 Statistical Analysis

#### 2.2.1 Model Specification

The SITAR model allows us to describe growth curves, particularly during puberty. It takes into account individual variability in growth patterns by allowing for the alignment of growth curves through three parameters: size, timing, and intensity. The SITAR model can be described as (Cole et al. 2010; Sandhu 2024):

$$\begin{array}{c} y_{ij}=\alpha_{0}+\alpha_{i}+\sum_{k=1}^{K}\beta_{k}h_{k}\left(\frac{x_{ij}-\bar{x}-(\;\zeta_{0}+\zeta_{i})}{e^{-(\gamma_{0}+\gamma_{i})}}\right)+\epsilon_{ij}\; \end{array}$$

where $y_{\mathrm{ij}}$ is the observed growth measurement for individual i at time j; $h_{k}(\;\cdot \;)$ are the natural cubic spline functions (k=1,...,K), and $\beta_{k}$ are the coefficients for the mean curve with $\alpha_{0}$, $\zeta_{0}$, and $\gamma_{0}$ as the population level average size, timing, and intensity parameters; $x_{\mathrm{ij}}$ is the age measurement for individual i at age j and $\bar{x}$ is the mean age of measurement; The individual‐specific group level effects (also referred to as random effects) for size ($\alpha_{i}$), timing ($\zeta_{i}$), and intensity ($\gamma_{i}$) describe how an individual's growth trajectory varies from the mean growth curve; and the $\epsilon_{ij}∼N(\;0,\sigma^{2})$ are the normally distributed residuals.

### 2.3 Prior Distributions

In Bayesian statistics, prior distributions express existing knowledge or assumptions about model parameters before observing the data. When fitting the SITAR model within a fully Bayesian framework (Sandhu 2024), it is necessary to specify a prior distribution for each parameter. In this study, we adopted the default weakly regularizing prior, which introduces modest constraints to reduce overfitting while remaining largely non‐informative. These priors are scaled to the data—specifically, to the standard deviations of the outcome and predictors—ensuring that they are appropriately adapted to the scale of the modeled variables.

### 2.4 Sensitivity Analysis

To maximize the use of available data, we evaluated the sensitivity of our model estimations to variations in the number of measurements per participant. We fitted the SITAR model separately for male and female subsamples, including only those with at least 11, 10, 9, 8, 7, 6, or 5 measurements, as well as a subsample using all available data.

### 2.5 Posterior Predictive Checks and Computation and Transparency

Boys and girls were analyzed separately. We used posterior predictive checks (Gabry et al. 2019). We used two chains for 6000 iterations with a warm‐up length of 3000 iterations to fit the models. The models were fitted using R statistical language (R Core Team 2024). The Bayesian methods were implemented using the bsitar package (Sandhu 2024), which uses the brms package (Bürkner 2017) to call Stan (Stan Development Team 2023).

### 2.6 Maturity Status

We used APHV, derived from the Bayesian SITAR models, as an indicator of maturational timing. To classify maturity status, we applied a distribution‐based approach using sex‐specific percentiles of the APHV estimates within our sample. Participants were categorized as early maturers (APHV below the 16th percentile), average maturers (APHV between the 16th and 84th percentiles), or late maturers (APHV above the 84th percentile). This percentile‐based categorization provides a practical means of representing inter‐individual differences in pubertal timing, and is commonly used to interpret maturity‐associated variation (Kozieł and Malina 2005; Malina et al. 2016; Tanner et al. 1966).

## 3 Results

Estimates from the Bayesian SITAR models remained consistent across different numbers of repeated measurements, indicating that measurement frequency had minimal influence on estimates of APHV and PHV. The standard deviation of the APHV estimates was 0.26 years for girls and 0.12 years for boys, while for PHV, standard deviations were 0.18 cm/year for girls and 0.44 cm/year for boys.

Posterior predictive checks, used to assess the model's ability to generate data resembling the observed values, demonstrated similarly effective performance across models with varying numbers of included measurements. Figure S1 (girls) and 2 (boys) present the kernel density estimates of the observed data alongside density estimates from datasets simulated from the posterior predictive distribution. The substantial overlap between observed and simulated distributions indicates that the models captured the underlying data structure well, providing a reasonable fit to the observed growth patterns in both sexes.

In girls, the mean APHV was 11.30 years (68% credible interval: 11.23 to 11.37), and the mean PHV was 8.38 cm/year (68% credible interval: 7.34 to 9.30). The age at takeoff growth velocity (ATGV) was estimated at 8.41 years (68% credible interval: 7.70 to 9.02), with a mean takeoff velocity (TGV) of 5.85 cm/year (68% credible interval: 5.10 to 6.50).

In boys, the mean APHV was 13.55 years (68% credible interval: 13.47 to 13.63), and the mean PHV was 9.52 cm/year (68% credible interval: 9.35 to 9.70). The ATGV was 11.19 years (68% credible interval: 11.06 to 11.32), and the mean TGV was 5.43 cm/year (68% credible interval: 4.79 to 6.04). A summary of the Bayesian SITAR model is presented as Supporting Information.

**FIGURE 1:**
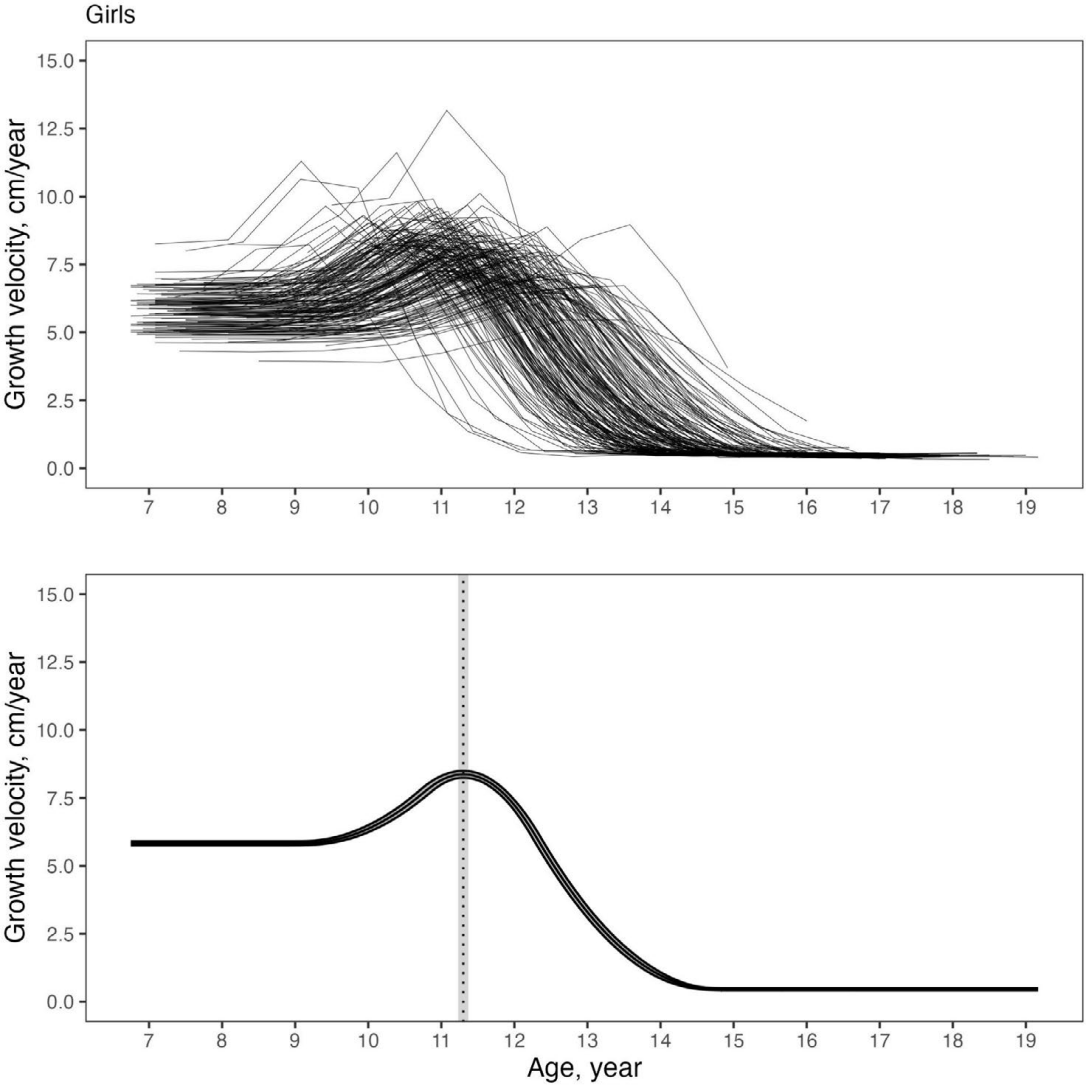
Growth velocity curve in cm/year for Brazilian girls. Bands represent the 68% credible intervals.

**FIGURE 2:**
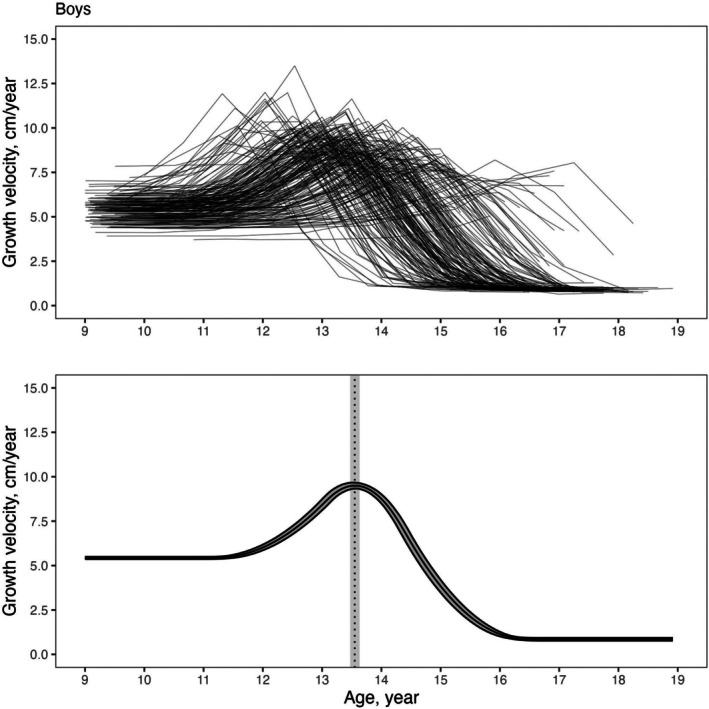
Growth velocity curve in cm/year for Brazilian boys. Bands represent the 68% credible intervals.

The fitted mean velocity curves and individual velocity curves are displayed in Figures 1 and 2 for girls and boys, respectively. The upper panels of these figures illustrate the substantial inter‐individual variation in PHV and APHV within both sexes.

Table 2 summarizes the growth velocities and ages at the puberty onset and peak growth in the three maturity groups of Brazilian girls and boys, respectively. For girls and boys of each sex, mean APHV and ATGV had higher values, while mean PHV and TGV had lower values across early, average, and late maturity groups.

**TABLE 2:** Means and 68% credible intervals for growth velocities and ages at puberty onset and peak height velocity in the three maturity groups of Brazilian girls and boys.

Measure	Group	Girls (*n* = 200)	Boys (*n* = 200)
ATGV, year	Early	8.01 (7.37; 8.33)	9.24 (8.86; 9.71)
	Average	8.29 (7.81; 8.80)	10.26 (9.72; 10.88)
	Late	9.11 (8.70; 9.50)	11.40 (10.96; 11.89)
TGV, cm/year	Early	6.18 (6.02; 6.75)	6.05 (5.34; 6.69)
	Average	5.93 (5.27; 6.56)	5.47 (4.93; 5.93)
	Late	5.17 (4.77; 5.72)	4.74 (4.33; 5.18)
APHV, year	Early	10.13 (9.88; 10.48)	12.11 (11.74; 12.50)
	Average	11.20 (10.74; 11.63)	13.38 (12.90; 13.87)
	Late	12.40 (12.09; 12.69)	14.99 (14.20; 15.70)
PHV, cm/year	Early	9.16 (8.59; 9.69)	10.53 (9.43; 11.72)
	Average	8.42 (7.53; 9.33)	9.50 (8.63; 10.40)
	Late	7.40 (6.83; 8.19)	8.28 (7.62; 9.09)

We examined the correlations between growth timing and tempo, specifically ATGV versus TGV and APHV versus PHV (Table S2). Both sexes showed moderate negative correlations in the full sample, indicating that earlier maturers tended to grow more rapidly (girls: APHV vs. PHV = −0.56; boys: −0.61). Patterns varied by maturity group: early maturing girls showed a strong negative correlation between ATGV and TGV (−0.68), while other groups displayed weaker or reversed associations. Among boys, the correlation between APHV and PHV remained consistently negative across maturity groups, whereas correlations between ATGV and TGV were more variable. These findings suggest that growth timing and tempo are interrelated, but their association differs by sex and maturity status.

## 4 Discussion

Using a Bayesian SITAR model, this study aimed to describe pubertal growth velocity curves in a sample of Brazilian children and adolescents. To our knowledge, this is one of the few longitudinal datasets of its kind in Brazil. While these findings offer valuable insights, they should be interpreted with caution due to Brazil's vast regional diversity in ancestry, socioeconomic status, and cultural background (Silva et al. 2012). Our sample, drawn from Florianópolis—a city with a high Human Development Index and a predominantly European‐descended population (Instituto Brasileiro de Geografia e Estatística—IBGE 2003, 2011)—provides a meaningful basis for comparison with both European longitudinal cohorts and regional Latin American data, particularly with respect to the timing and intensity of pubertal growth.

As expected, clear sex differences were observed in the timing and intensity of the adolescent growth spurt. The estimated mean APHV was 11.3 years for girls and 13.5 years for boys. These values were slightly earlier than those reported in classic longitudinal studies from the 20th century, such as the Fels, Harpenden, and Leuven cohorts (Beunen et al. 1994; Roche 1992; Tanner et al. 1966), which typically placed APHV between 11.4 and 12.2 years in girls and between 13.4 and 14.4 years in boys. Our findings also aligned closely with the large‐scale ALSPAC study, which used SITAR modeling and estimated APHV at 11.7 years for girls and 13.5 years for boys (Cole 2020). The ALSPAC cohort, recruited in the early 1990s and followed into young adulthood (Boyd et al. 2013), offers a valuable contemporary comparison. Similarly, our estimated PHV values were modestly lower than those from historical studies and ALSPAC (Cole 2020; Frysz et al. 2018; Malina et al. 1988), reinforcing the notion of a secular trend toward earlier pubertal growth, likely influenced by improvements in nutrition, health, and living conditions (Cole 2000).

Our findings contribute to a growing body of evidence on pubertal development in Latin America. In Brazil, the Cariri Healthy Growth Study offers one of the few available references (Guimarães et al. 2020; Silva et al. 2019), although it relied on mixed‐longitudinal data and focused primarily on physical performance during adolescence. Studies from other Latin American countries also support considerable variability in APHV. For example, in Venezuelan adolescent girls it has been reported APHV estimates of approximately 11.7 years for girls and 13.5 years for boys using cubic spline function (Lopez‐Blanco et al. 1995). Guatemalan Ladino children—whose ancestry is partially European but who experience markedly different environmental conditions—showed later APHV, with estimates of around 12.0 years in girls and 13.6 years in boys using the Preece‐Baines model I function (Bogin et al. 1992). Together, these comparisons highlight the influence of ancestry, socioeconomic context, and environmental exposures on pubertal timing across Latin America, and reinforce the importance of developing region‐specific growth references.

While broadly consistent with contemporary estimates, the earlier APHV and lower PHV values observed in our sample likely reflect a combination of secular trends and sample‐specific characteristics. The relatively high socioeconomic status and European ancestry of participants in Florianópolis may partially explain these patterns, as similar profiles are often associated with earlier growth spurts. Importantly, methodological differences across studies likely contribute to discrepancies in APHV estimates (Boeyer et al. 2020; Simpkin et al. 2017). Some studies rely on visual inspection or parametric curve fitting (e.g., Preece‐Baines or polynomial functions), while others, like ours and ALSPAC, use SITAR modeling. Our application of a Bayesian version of the SITAR model offers additional sensitivity to individual growth trajectories, potentially capturing growth inflection points earlier than traditional methods. This modeling approach, together with our classification of maturity groups based on standard deviation thresholds rather than percentiles, may contribute to the earlier APHV values observed in our sample, especially among girls.

A key finding was the considerable inter‐individual variability in growth timing and tempo. Using maturity group classifications based on deviations from the sample mean APHV, we observed meaningful differences across early, average, and late maturers. In girls, earlier maturation was particularly associated with a more rapid growth tempo. Early‐maturing girls showed the strongest association between early takeoff and faster peak growth, while boys displayed a more consistent timing–tempo relationship across groups. These findings are consistent with previous evidence that female pubertal development is more variable and sensitive to social and environmental factors (Parent et al. 2003).

Further comparisons with the ALSPAC cohort provide additional insight. Among early‐maturing girls, the age at takeoff and APHV in our sample were 8.0 and 10.1 years, respectively, slightly earlier than the 8.5–8.7 and 10.0–11.1 years reported in ALSPAC (Cole 2020). For late‐maturing girls, our sample showed ATGV and APHV at 9.1 and 12.4 years, respectively, while ALSPAC reported ranges from 9.4 to 11.1 years for takeoff and 12.7 to 15.2 years for APHV. These differences likely reflect a combination of secular, socioeconomic, and methodological influences. In contrast, boys in our study exhibited ATGV and APHV estimates that were more similar to those reported in ALSPAC, suggesting that sex‐specific developmental dynamics may also play a role in moderating inter‐cohort variability.

From a practical perspective, the observed variability in pubertal timing has meaningful implications. In settings such as youth sport, education, and public health, differences in biological maturity can influence participation, performance, and injury risk (Carvalho and Gonçalves 2023; Lloyd et al. 2015). Our findings reinforce the value of accounting for maturity status in interpreting growth patterns and developmental progress during adolescence. Group‐based analyses, while informative, may obscure meaningful individual variation that is essential for personalized monitoring and intervention.

A notable strength of this study is the use of a Bayesian SITAR model, which estimates individual growth trajectories while capturing population‐level trends. Unlike traditional approaches that treat APHV and PHV as fixed parameters, SITAR accommodates nonlinear dynamics and individual heterogeneity. The Bayesian framework further enhances model robustness by integrating prior knowledge and providing full uncertainty quantification—advantages that are particularly important in real‐world longitudinal datasets with irregular intervals or missing data (Gelman 2003; van de Schoot et al. 2014). Posterior predictive checks and sensitivity analyses (see Supporting Information) supported the validity of our growth estimates and demonstrated model stability under different prior assumptions and measurement variability (Gabry et al. 2019; Gelman et al. 2020).

Nevertheless, some limitations should be noted. While the sample reflects certain socioeconomic and ethnic patterns typical of southern Brazil, it is not nationally representative. Neither ancestry nor socioeconomic status was directly assessed, which restricts our ability to formally test their associations with pubertal timing. As such, caution is needed in generalizing these findings to populations with different demographic profiles or living conditions. Additional longitudinal studies in other Brazilian regions and among more socioeconomically diverse populations are essential to refine growth references and expand their relevance for health monitoring and developmental research.

## 5 Conclusion

The Bayesian SITAR model effectively captured individual growth trajectories and inter‐individual variation in pubertal timing among Brazilian youth. Our findings suggest that both the onset of the adolescent growth spurt and the age at which peak growth in stature occurs are earlier than typically reported, likely influenced by factors such as socioeconomic status and ancestry. Notably, we observed considerable variability in growth timing, particularly among girls, with both early and late maturers reaching their peak growth earlier than expected. This highlights the importance of considering the full range of maturity‐related differences when interpreting developmental patterns during adolescence.

Individualized growth estimates provide valuable benchmarks for clinicians and educators, facilitating the early identification of potential health or developmental concerns. Expanding this research to more socioeconomically and ancestrally diverse populations may enhance the applicability of growth references and improve the relevance of pubertal growth standards across varied demographic contexts.

## Author Contributions

**Luciano G. Galvão:** investigation, data curation, writing – original draft preparation. **Fábio C. Karasiak:** investigation, writing – original draft preparation. **Victor J.S. Conceição:** investigation, writing – review and editing. **Diego Augusto Santos Silva:** conceptualization, writing – review and editing. **Humberto M. Carvalho:** conceptualization, formal analysis, writing – review and editing.

## Conflicts of Interest

The authors declare no conflicts of interest.

## Supporting information


**Data S1:** Supporting Information.

## Data Availability

The data that support the findings of this study are available on request from the corresponding author. The data are not publicly available due to privacy or ethical restrictions.
