# Supplementary material for: Modeling Pubertal Growth Variability in Schoolchildren From Florianópolis, Santa Catarina, Brazil: A Bayesian Analysis
Source: Am J Hum Biol. 2025 Aug 29;37(9):e70129. doi: 10.1002/ajhb.70129 (PMC12395401; doi:10.1002/ajhb.70129)
Supplement: Supplementary file 1 — Data S1: Supporting Information. [file AJHB-37-e70129-s001.pdf]

Supplementary Material for the Manuscript: "Modeling Pubertal Growth Variability in Schoolchildren from Florianópolis, Santa Catarina, Brazil: A Bayesian Analysis"

Luciano G. Galvão[1], Fábio C. Karasiak[1], Victor J. S. Conceição[2], Diego Augusto Santos Silva[1], Humberto M. Carvalho[1]

[1] School of Sports, Federal University of Santa Catarina, Brazil [2] Laboratory School, School of Education, Federal University of Santa Catarina, Brazil

This supplementary file provides additional information supporting the results presented in the main manuscript titled *"Modeling Pubertal Growth Variability in Schoolchildren from Florianópolis, Santa Catarina, Brazil: A Bayesian Analysis."* It includes model diagnostics, sensitivity analyses, and detailed summaries of model parameters.

Specifically, we present:

- The distribution of repeated anthropometric measurements per participant (Supplementary Figure 1);
- Posterior predictive checks comparing observed data with simulations from the Bayesian SITAR model (Supplementary Figures 2 and 3);
- A summary of population-level model estimates (Supplementary Table 1);
- Correlation estimates between timing and tempo of pubertal growth, stratified by sex and maturity group (Supplementary Table 2).

These materials complement the main findings by demonstrating model adequacy, estimation stability, and individual-level variation in pubertal growth patterns.

Supplementary Figures and Tables

To ensure sufficient longitudinal coverage of the pubertal growth period, inclusion criteria required at least four repeated measurements between ages 9.9 and 14.0 years for girls, and between 10.9 and 15.0 years for boys. The distribution of measurements per participant is shown in Supplementary Figure 1.

To evaluate model fit, we used posterior predictive checks. Supplementary Figures 2 (girls) and 3 (boys) compare kernel density estimates of the observed data with those from data simulated from the posterior predictive distribution. These checks demonstrate that the Bayesian SITAR model provided an adequate fit to the observed data in both sexes, with substantial overlap between observed and simulated distributions.

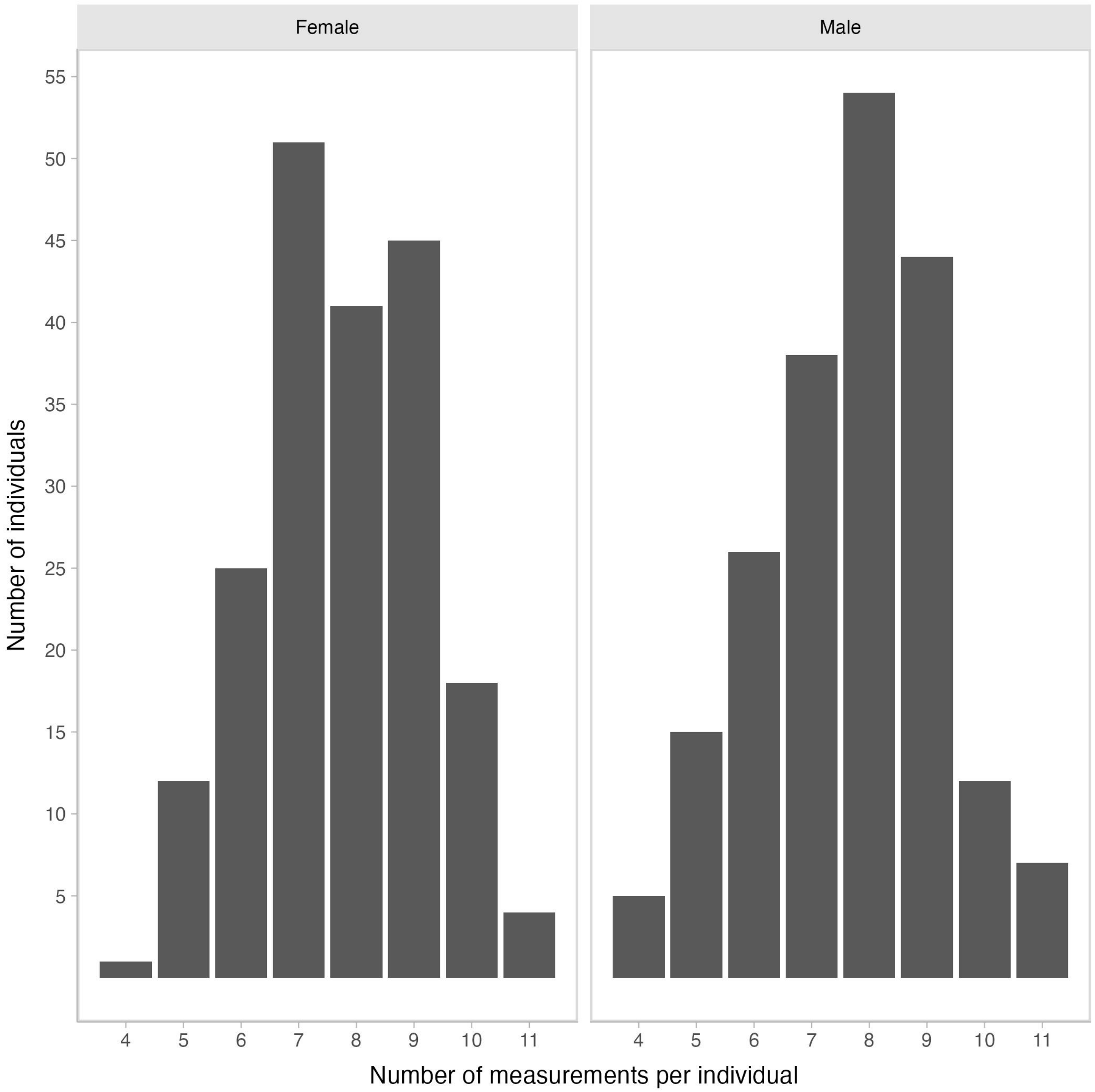

Supplementary figure 1: Distribution of the number of measurements per participant.

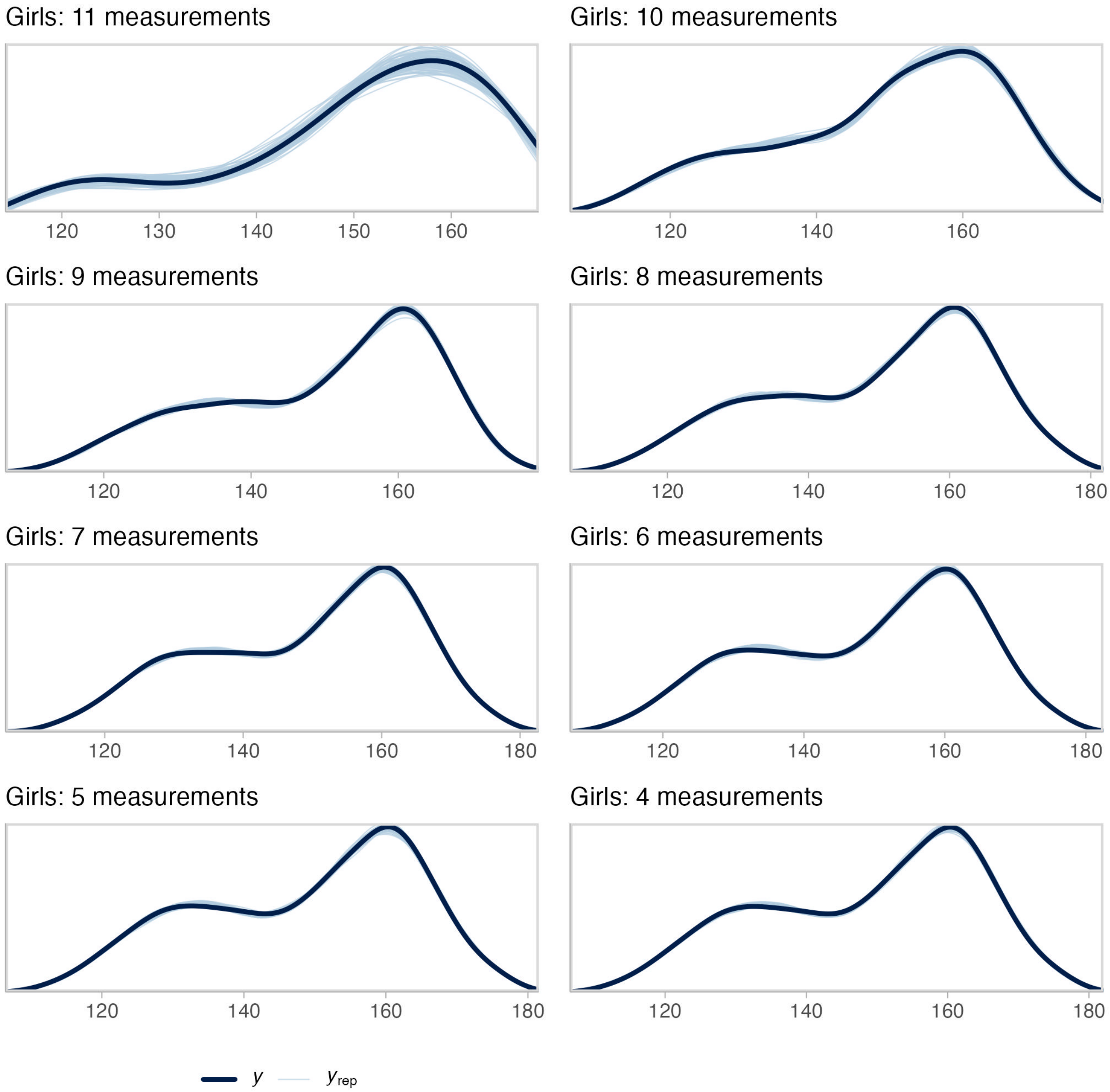

Supplementary figure 2: Kernel density estimate of the observed data set y, with density estimates for simulated data sets yrep drawn from the posterior predictive distribution (girls).

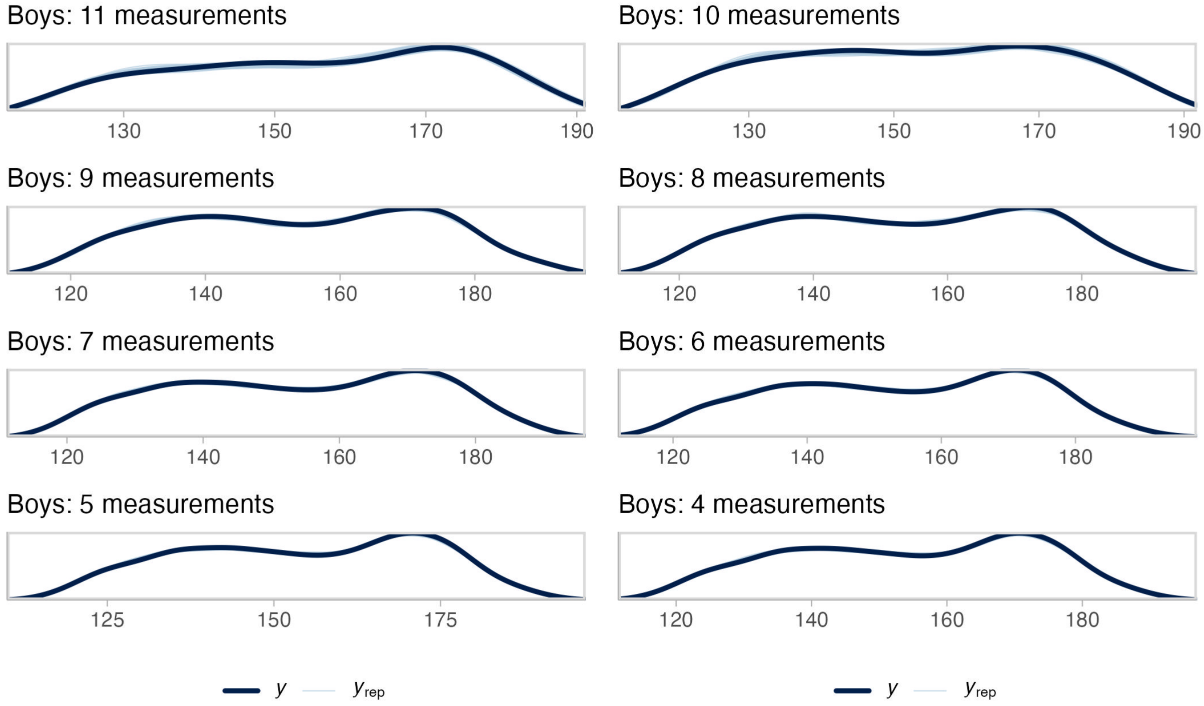

Supplementary figure 3: Kernel density estimate of the observed data set y, with density estimates for simulated data sets yrep drawn from the posterior predictive distribution (boys).

The supplementary table 1 summarizes the standard deviations (with 68% credible intervals) of the population-level SITAR parameters: size (stature), timing (horizontal shift), and intensity (scale of the velocity curve), along with residual variability. Boys showed slightly more variability in timing and residuals, suggesting greater heterogeneity in their growth patterns.

Supplementary table 1: Standard deviations (68% credible intervals) of SITAR model parameters and residuals, by sex.

	Girls (n = 197)	Boys (n = 297)
Stature, cm	6.47 (6.17; 6.79)	6.42 (6.07; 6.77)
Timing, fractional	0.79 (0.75; 0.83)	1.07 (1.00; 1.13)
Intensity, fractional	0.13 (0.13; 0.13)	0.13 (0.12; 0.13)
Residual, cm	1.00 (0.98; 1.03)	1.15 (1.12; 1.17)

Supplementary table 2 presents Pearson correlation coefficients (with 68% credible intervals) for two timing indicators—Age at Take-Off (ATGV) and Age at Peak Height Velocity (APHV)—and two tempo indicators—Take-Off Growth Velocity (TGV) and Peak Height Velocity (PHV). Results are shown for the full sample and by maturity group. Earlier timing was generally associated with greater growth tempo, although strength and direction of associations varied by group, particularly among girls.

Supplementary table 2: Pearson correlations (68% credible intervals) between growth timing and tempo, by sex and maturity group. Negative values suggest that earlier maturing individuals tend to grow faster.

		ATGV vs. TGV	APHV vs. PHV
**Girls**			
	All sample	-0.30 (-0.37; -0.22)	-0.56 (-0.62; -0.50)
	Early	-0.68 (-0.79; -0.56)	-0.20 (-0.28; -0.12)
	Average	0.51 (0.43; 0.60)	-0.01 (-0.04; 0.02)
	Late	0.30 (0.18; 0.42)	-0.28 (-0.36; -0.20)
**Boys**			
	All sample	-0.32 (-0.38; -0.25)	-0.61 (-0.66; -0.55)
	Early	0.07 (-0.01; 0.16)	-0.17 (-0.23; -0.10)
	Average	0.31 (0.24; 0.39)	-0.07 (-0.10; -0.04)
	Late	-0.17 (-0.27; -0.06)	-0.50 (-0.57; -0.43)
